# Lectures on Subjects Connected with Clinical Medicine, Comprising Diseases of the Heart

**Published:** 1847-01

**Authors:** 


### Art. XIII.

*Lectures on Subjects connected with Clinical Medicine, comprising Diseases of the Heart.* By P. M. Latham, m.d., &c. In Two Volumes. Vol. II.—*London,* 1846. Small 8vo, pp. 419.

Some time since it became our pleasing duty to place before the readers of this Journal* an outline of the facts, doctrines, and opinions which Dr. Latham had put forth in a first volume of Lectures, clinical in their main character, on Diseases of the Heart. In the article then devoted to the subject, we examined somewhat in detail such of the author's general principles of medical faith as we deemed necessary; we shall consequently be enabled to devote the present sketch altogether to particulars.

Lecture XVIII.—Here Dr. Latham speaks concerning that period in the course of endocardial and of pericardial inflammations which intervenes between the cessation of all acute evidences of disease and the restitution of the organ to a state compatible with existing safety. Of ninety cases of acute rheumatism in which the endocardium or pericardium or both were inflamed, three (in two both membranes were implicated) terminated by death. In seventeen instances only could the observer "feel anything like an assurance of perfect recovery." And the remaining seventy patients? what of them? Why they left the hospital more or less completely free from functional disturbance of the heart—such disturbance as directly and distinctly affected their comfort and was perceptible to themselves—but carrying away with them that significant murmur, the sure index to imperfectly repaired mischief within the heart. But they were safe for the present; a process of reparation of some kind had been established, and upon this process or this condition Dr. Latham wisely comments as follows:

* See Brit. and For. Med. Rev., July, 1845.

"But what is reparation? It is neither health nor disease; but it stands midway between them, and partakes of the nature of both. Now it is nearer to one, and now to the other; now ready to fall back into disease, and now to go forward into health. Truly this reparation demands as much of the physician's care as either of the other two; for it has its aids and its hinderances, which it is our business to study and to interpret; its aids, that we may apply them and cherish them, and in every way make the most of them; its hinderances, that we may intercept them, or lessen them, or counteract or annul them altogether." (p. 5.)

Seldom to endocarditis, oftener to pericarditis, and oftener to endo-pericarditis there may succeed a time—days or weeks—during which it is yet matter of question whether the individual will live or die. The most intelligible cause of this is, Dr. Latham suggests, the variable amount of injury which has accrued to the heart by the inflammation that has passed. When there is a loud endocardial murmur, for instance, and no other condition implying disorder of the heart, no palpitation, no præcordial anxiety or pain, no œdema of the ankles, "surely *there is reason to believe* that the damage is small, consisting in those little beads of lymph, deposited upon the free edge of a valve, which dissection discloses as the effect of acute inflammation." But an endocardial murmur may be attended with the functional disturbances, which we have just supposed to be absent, and with others; and here Dr. Latham would *believe* that a greater amount of anatomical change has occurred. For acute endocarditis

"Can do more than deposit specks of lymph upon the edges of a valve; it can spread a layer of lymph over a great superficial extent, even (as I have seen) throughout an entire auricle; it can accumulate masses of lymph as large as a pea or a cherry-stone, or larger, and leave them pendulous into the cavity of the ventricle; or it can destroy half a valve by ulceration, or carry away a long strip of the membrane, and lay bare the muscular substance; or (as I have known) it can penetrate from one auricle to the other, and lay them both together. Here are some forms of injury too destructive to admit any such degree of reparation as will allow life to go on at all; and here are others not destructive enough to make such reparation altogether impossible, but only capable of it slowly, and likely to keep life in jeopardy until it is accomplished." (p. 8.)

Now the doctrine taught by this antithesis may or may not be correct in respect of individual cases of cardiac disease; but it is certainly incorrect as a general doctrine of pathology (the pathological anatomists know the fact, and strive, but ineffectually, to weaken its force). There is, in truth, no constant or tolerably constant ratio between the amount of anatomical change in organs and the extent to which their functions are perverted; the lungs, the brain, the kidneys, the intestine, furnish daily examples of the total deficiency of harmony of (what may be termed) the anatomico-physiological kind. Hence it is that although, in distinguishing the nature of a disease, the most important point is the due cognizance of anatomical alteration, in prognosticating its issue and its course, the general condition of the individual plus the local imperfection of function furnishes the most pertinent and faithful clue. But Dr. Latham spoke of the heart; and of the heart our proposition is equally true. However we did not quote the passage just set down, because of its pathological doctrine alone, but because also of the inkling it gives of Dr. Latham's experience in the morbid changes produced by acute endocarditis; certain of these changes are assuredly remarkable, and as assuredly infinitely rare.

But here is a passage, striking in the manner of Dr. Latham, and occurring a few pages on, which corroborates, to our very heart's content, the justness of the criticism now ventured upon :

"Look to the mere matter and bulk of things, and think only of what is visible, tangible, and audible in parts, and you will come to strange conclusions: you will see people die of too little to kill them, and see people survive what is enough to kill them twenty times over. But if, in such events, you would know what it is that *mainly* kills, and what that *mainly* saves, you must look out of the part into the constitution at large: you must do so especially in diseases of the heart." (p. 14.)

The lecture closes with an allusion—it is a mere allusion—to certain cases in which life is jeopardized, not by the cardiac disease in itself, not by the general constitutional pravity of the individual, but by certain disordered conditions of the brain and spinal cord (maniacal delirium, epileptic or tetanic convulsions, chorea, coma, fatuity) superinduced by the cardiac suffering. And hereupon Dr. Latham takes just occasion to observe that disease is "a great physiological teacher,"—he has "never laid bare a living brain, a living spinal marrow, or a living heart,"—"he has read of experiments which he has never performed, and *never could bear to see;*"—but of little importance is the loss or the deficiency in the present case. For, "have all the experiments that were ever done or perpetrated upon living animals given intimation of an influence like this, proceeding from the heart to the brain, and from the heart to the spinal marrow? Has not disease here been our teacher?" True in science, true in morality, true in religion! Yes, disease is *the* physiological teacher. How admirably, for instance, has disease of the brain demonstrated the fallacy of the localizations of various mental and affective faculties, as set down by the reckless perpetrators of aimless and barbarous vivisections! How emphatically were we told by the hewers of living flesh, that the anterior lobes preside over the function of speech; that the force regulating movements held its seat in the cerebellum; that loss of motive power in the upper extremity would follow implication of the optic thalamus, of the lower extremity injury to the corpus striatum;—and how much of this has clinical experience proved to be real? Not one jot, not one tittle! Far from bearing out the phantasies of these pseudo-philosophers (whose wisdom consists in the power to invent new methods of torture, and the stoicism to survey with indifference the writhings of their victims), Nature, watched clinically, is seen, as it were, to tax her ingenuity in overturning them—so various, so unmistakeable, and so perpetual are the contradictions she furnishes to the inferences founded on the results of vivisection. There is no science, and no true benefit to humanity in these practices, which simply confer notoriety on those who pursue them, and apparently—but not really—save our generation the labour of clinical observation. We are glad to find Dr. Latham has the courage to confess he can feel for the sufferings of the lower animals, and the honesty to admit his want of sympathy with the *hoc genus omne* of nerve-twitchers and brain-cutters; he "*may* have learned something from them; something, how dearly purchased!"

LECTURE XIX. "It is a general truth," observes Dr. Latham, "never formally declared, perhaps, but well worth our notice, and of great practical importance, that organs must be previously sound to show clearly the nature of the injury or malady which they suffer, and that in proportion as they are unsound, they are spoiled for giving expression to the ills which afterwards befall them........A broken instrument is ever out of tune; whatever key you touch you can never bring out the right note corresponding with it." (p. 32.) This is perfectly true, and it is true not only in respect of functional disturbances, but also of physical signs. The history of the physical signs of the membranous inflammations of the heart proves this; but that history does not, in our experience, allow the clinical observer to acquiesce in the dictum of Dr. Latham: "In the first inflammation of the sound heart they were everything; in all after inflammations of the unsound heart *they are nothing.*" And then proceeding to particulars, Dr. Latham affirms that in after inflammations of the pericardium there is no pericardial murmur, "and none there can be if its surfaces adhere completely. And if they adhere partially, and there be a murmur, it will not have the proper attrition in it, and so will want the exocardial (pericardial) character." Now we cannot help suspecting that Dr. Latham's devotion to antithesis has led him to state his doctrine of the inutility of physical signs in a second inflammation of the pericardium both more broadly and more uncompromisingly than his own observation, had he accurately analysed *all* its results, would warrant him in doing. It is no doubt true, that *if* the pericardium be totally adherent to the heart from previous inflammation, new inflammation will set up no friction-sound on its surface; but such total adhesion is not the common result of pericarditis. Imperfect adhesion is the rule; hence Dr. Latham must turn for defence of his general proposition to the deficiency of "the proper attrition" in the new-made sound by the new-made pericarditis. But admitting (which is not always, we say, admissible, except *argumenti gratiâ*) that the attrition character is wanting—the position in which the murmur is heard, its instability in character as in precise locality, its non-transmission beyond the præcordial region, its evident superficial seat, all these conditions will point to the pericardium as the seat of the new-made sound. A case which we have at present under observation illustrates and justifies these remarks; but we must confess we know not (nor does any one know, nor can any one now guess) in what *proportion* of cases similar conditions of murmur may be verified and *new* pericarditis be diagnosticated.

Next Dr. Latham applies his general doctrine to endocarditis. It is well known to every one accustomed to pass round the wards of hospitals, that it is the general habit, on the admission of a patient with acute rheumatism, to pronounce that patient to be the subject of acute endocarditis, if an endocardial murmur exist. Yet observe how erroneous the conclusion; for the murmur audible may be either the result of old endocarditis, or of new endocarditis, or of new endocarditis superadded to old, and both conducing to the same modified sound. All this Dr. Latham puts with his usual point and terseness in the page before us; and the fact that he does so, supplies a very remarkable illustration of the indubitable truth in regard of the mechanism of mind, that multitudes of men will have a keen perception of, and will emphatically uphold, a certain given general doctrine as such, and yet fail altogether to see its applicability, or, at least, to apply it, in particular cases. And the fact supplies the illustration in this wise: in Dr. Latham's first volume appear tabular views of the frequency of membranous inflammations of the heart in rheumatism, and in those tables, and in the commentary upon them, all the cases of endocarditis are referred to as examples of *acute* inflammation. This matter has been better elucidated by Dr. Taylor, in his most valuable paper on the 'Causes of Pericarditis,'* than by any other writer, though within certain limits his observations have been anticipated by the statements of M. Chomel.

But the great practical question is this,—are there any means whereby the recent can be distinguished from the old endocarditis, and the compound of both diseases from either singly? So long as all cases of rheumatic endocarditis, were habitually, when observed, set down as recent, it was not to be expected that any clue to the problem should be discovered,—its necessity was not dreamed of, why then should it be looked for? Yet the point is a peculiarly interesting one; a man lies before you, his joints the seat of migratory rheumatism, his heart excited, an endocardial murmur audible. Are you to forget his rheumatism, comparatively speaking, and treat his heart, or give yourself no special present anxiety concerning the state of that organ? The answer must depend upon your power to diagnosticate the age of the endocarditis. Now, how far goes that power? Dr. Taylor has a claim to be heard on this question, for his observations upon it are not only true and just, but stamped (like his entire paper) with a character of philosophical caution and severity, which is as striking as it is unfortunately rare in medical writings. He says:

"I have been enabled to discover the existence of *recent* endocarditis in a few cases, chiefly by the following circumstances: 1. By the appearance of a bellows-murmur, which could not be ascribed to rheumatism, or to any other obvious cause; and in a patient who was found to be free from it on admission. 2. By some considerable change in the rhythm of the heart, in cases in which it was at first found to be natural. I have observed the pulse to become *decidedly less frequent*, and each individual beat to be more slowly performed; in other words, both *slow* and *tardy*. The action of the heart becomes irregular, both as to its force and rapidity, and the duration of the periods of rest and often also, intermitting. With these signs, I have generally observed the bellows-murmur to persist after the heart's action has again become regular. We might not perhaps *a priori* have expected the contractions of the heart to become less frequent and tardy in a disease which must increase the sensibility of its lining membrane. Perhaps the cause is the same as that of suspension of the peristaltic action of the intestines in peritonitis, and as that of the paralysis of the intercostal muscles, which some physicians believe to occur in pleuritis. Be the explanation what it may, however, I am quite sure, both from the careful examination of such cases during life, and from an examination of the heart after death, that the symptoms I have described are produced by endocarditis. But then they are not present in all cases of endocarditis, as I have also ascertained. Were I to trust to the impression on my own mind, unchecked by figures, I should say that I have found the symptoms in question, in cases of endocarditis, unaccompanied by pericarditis; and that in cases of endo-pericarditis, I have found the frequency and perhaps the quickness of the pulse increased, with or without intermittent or irregular action. Cases of pericarditis and of endo-pericarditis, with increased frequency and quickness of pulse, would appear to be unfavorable to the explanation of the slowness and tardiness of the pulse already adverted to." (loc. cit., p. 496.)

But it will be perceived that, whatever be the accuracy of these observations, they have no reference to the solution of the problem most frequently presented in actual practice: they refer to cases in which an

* Med.-Chir. Transactions, vol. xxviii, p. 493.

endocardial murmur is developed *after* the patient has come under observation, and not to cases in which it is already existent when the physician first sees him.

Lecture XX. But, as is well known, organs which have once suffered inflammation are prone to suffer afresh under the influence of exciting causes. When a man has had his cardiac membranes once affected, and is subsequently seized with rheumatism, pneumonia, pleurisy, or fever, and exhibits increase of habitual palpitation and pain, then the existence of fresh inflammation should, according to Dr. Latham, "be assumed as a fact." He goes on to say, that, for admitting the existence of this new inflammation, and for our guidance in treating it, we "have only the warrant of conjecture. . . . . But there is such a thing as sober conjecture, as well as sober certainty. And diseases are treated, and cures are achieved, and lives are saved, *as often under the guidance of one as the other.*" This seems a startling article of therapeutical faith, but it nevertheless conforms with fact, and will be found to lose much of its paradoxical character in the commentary of its promulgator.

Lecture XXI. The lecturer proceeds to the consideration of the unrepaired effects of endocarditis and pericarditis as constituting permanent unsoundness of the heart in themselves, and becoming the possible elements of further unsoundness beyond themselves. The morbid appearances left by endocarditis he finds to be "opacity and thickening of the membrane, marks of perfect and imperfect cicatrization, and breach of surface or solution of continuity." Now, concerning the real existence of the first kind of change, no doubt can be entertained, but of the second and third we are not so sure. Hear, however, the author's description:

"Beside such general opacity and thickening a particular valve sometimes presents a hard elevated line or ridge where it is especially thickened, or a small spot where it is indented or depressed, looking like a complete cicatrization in one case, and an incomplete cicatrization in the other. Sometimes a valve is perforated or cribriform or it wants a portion at its edge, or a tendinous cord is snapt in two, and its ends are hanging loose within the cavity of the ventricle." (p. 79.)

Now, we altogether question the fact of the appearances here referred to signifying a cicatrization-process, either perfect or imperfect: we know the appearances, and we cannot conceive them to mean anything but irregularity in the deposition of the plastic matter exuded by the inflammation which is gone. Why, if these be signs of cicatrization, ulceration and healing of the valves are among the commonest of phenomena,—and yet, who is in the habit of stumbling upon many unquestionable valvular ulcers? And, further, the perforations and cribriform appearances of the valves, which are doubtless far from uncommon, cannot, with any colour of plausibility, as far as we know, be traced to inflammatory action. What is the coexistent change in the anatomy of the parts, what the feature or features in the bygone clinical history of the owner of those parts, which enables Dr. Latham to ascribe, thus unhesitatingly, the cribriform condition of valves to inflammation? We know of none, and shall be glad to learn what they are.

Lecture XXII. Here are considered the consequences to life and health produced by the permanent unsoundness entailed by endocarditis. There are three kinds of cases observed, and the sum of Dr. Latham's observations may be given in the following quotation, wherein well-known facts are impressively and pointedly put:

"Taking then the three descriptions of cases in their order, I believe it to be the tendency of each to pass progressively onward into the others. The endocardial murmur left by acute endocarditis may be simple and alone, and so it may remain for years, but it is ever apt to have a palpitation added to it. The palpitation accompanying the murmur may be occasional only, and so it may continue for years; but in the mean time, it is ever ready to become permanent. The permanent palpitation may remain for a while moderate in degree, but it is always tending to become greater and greater. Of these three conditions, then, the best that experience allows us to hope is, that each may remain stationary: for their changes are never retrograde, but always progressive, and always for the worse. Each condition becomes worse as it is converted into the other, and the condition of permanent palpitation passes on to new results, and to the final and fatal event." (pp. 96-7.)

Lecture XXIII. In this discourse the permanent unsoundness derived from pericarditis is the subject. Once pericarditis has ceased, auscultatory signs cannot be appealed to for information concerning the state of the pericardium. There is, in the first place, no such thing known, Dr. Latham "believes," as permanent continuance of a pericardial murmur. In this belief Dr. Latham is certainly supported by all recorded experience, so far as we know; but we have our *suspicions* (and why suspicions may long continue suspicions only is readily intelligible in a case like this), that there are *rare* instances in which the friction-sound of pericarditis permanently remains in a modified form.

There may be, as results of pericarditis—1, universal adhesion of the pericardium, and complete obliteration of its cavity by hardly any apparent medium of adventitious substance; or, 2, instead of the adhesion of the pericardium being total and the obliteration of the cavity complete, both may be partial only; and, 3, it is not only the *extent* of adhesion that varies, but the *quantity* of uniting medium also. There may be naught but thready filamentous texture holding the two surfaces together, or there may be a solid mass, more than an inch in thickness, and this solid mass is well described as follows:

"Its texture sometimes laminated like the coagulum of an aneurismal sac, red or tawny near the heart, and pale or white more remote from it, sometimes of a mixed consistence, in part almost liquid and purulent, and in part solid or tuberculous. Or the adventitious substance has been of one uniform texture, either so like muscle as to be at first mistaken for the fleshy substance of the heart itself, or so far firmer than muscle as to resemble flesh hardened in brine, either much paler than the heart, or much redder from being deeply injected with blood. This tough flesh-like substance may occupy a portion only of the surface of the heart or the whole of it. I have seen it opposite the right auricle, while everywhere else the pericardium has closely adhered with little intervening medium, and I have seen it enveloping the entire organ and forming round it (as it were) another case of muscle. And then, if (what often happens) the muscular substance of the heart itself be augmented, a strange spectacle is disclosed on dissection. There is an enormous mass displacing the lungs and leaving nothing visible in the entire front of the chest but itself." (pp. 115-16.)

Lecture XXIV. "Permanent unsoundness of the endocardium from diseases of a specific and malignant nature, especially from analogous formations," forms the subject of this lecture,—one of the least satisfactory of the set. We do not see how "analogous formations" (e. g. ossiform, cartilaginiform matter, &c.) can be classed as malignant. And we believe that Dr. Latham is not justified in calling the cartilaginiform-looking productions which occur in connexion with the membranes "cartilaginous depositions,"—they have not the intimate structure of cartilage. The diseases giving rise to these depositions, Dr. Latham assumes to be different from inflammation—whereby he means, different in their origin from that state. Now, this may be so,—it may be that ossiform-deposition takes place where no inflammation has gone before, but Dr. Latham should have proved this, as there are persons who hold the opposite opinion. M. Bizot has clearly demonstrated the fact that in the arteries the formation of ossiform substance arises independently of inflammatory processes, but it does not thence follow as a necessary consequence that they shall have no connexion therewith in the heart.

LECTURE XXV. There is nothing novel in the matter of this lecture, which is devoted to the consideration of "acute, pervasive, pus-depositing inflammation" of the muscular substance of the heart. The cases referred to are those of Messrs. Stanley and Salter: they are said to be the only ones that have fallen within the lecturer's knowledge.

LECTURE XXVI. Chronic inflammation of the substance of the heart, terminating in ulceration, in partial dilatation, or in possible rupture, is the main subject of consideration in this lecture. Cardiac aneurism receives no addition to its pathology at the hands of the author; and, like his predecessors, he is obliged to admit that the diagnosis of the affection is unattainable during life. This admission he makes in the following passage, which gives a good notion of the existing deficiency of our knowledge on the subject:

"Now our clinical acquaintance with these diseases during life has not kept pace with the knowledge which anatomical investigation has procured us of them after death. Sometimes they have had their beginning and their progress without awakening in the patient the least suspicion of anything wrong within the heart. He has had no consciousness of ailment or suffering, and the fatal consummation has been an awful surprise. Sometimes they have been attended with suffering enough to alarm the patient, and by symptoms enough to enable the physician to infer damage of the heart, and even to anticipate its fatal event, but not to be sure of its nature; such as faltering and failure of the circulation and dyspnœa and anguish, either constant with occasional aggravations, or altogether occasional and in paroxysms, but, whether constant or occasional, never attended with any precise auscultatory signs. But sometimes they have had the accompaniment withal of precise auscultatory signs, and these have gone to the clear diagnosis of certain present conditions of disorganization within the heart. But then these conditions have been no essential part of *the* disease. Auscultation has told of hypertrophy and general dilatation of the ventricle with certainty enough, but it has left the partial aneurismal dilatation and the circumscribed progressive ulceration and the impending rupture entirely unsuspected." (pp. 148-9.)

Hereupon follow two cases in which the disease existed, but was not even dreamed of during life. The narratives of these cases are instructive, but their utility does not appear to us as much increased as the lecturer, from internal evidence, probably fancies, by the quaint and rather affected style in which they are set down. It strikes us that, if we heard a lecturer proceed to the description of a case of disease in the following manner (as Dr. Latham does in another part of his volume)—"There was a certain youth, David Aikin by name, and he was fifteen years of age; he was a poor puny lad, and first came under my care at St. Bartholomew's Hospital," &c.—we should feel disposed rather to smile in expectation of a jocular tale, than to put our wits in order for the reception of solid instruction. But this is, we confess, little more than a matter of taste.

And of the evidence of fatty degeneration of the heart Dr. Latham has little to say; but he appears to us to have said that little so well, that we extract the passage;—it contains the sum of knowledge on the matter:

"During the life of the patient, however, there is (as far as I know) no sure diagnosis of the fat heart, but a probable conjecture only. And even this probable conjecture can scarcely be made while the heart is *simply* fat, and nothing more, but must wait until it has reached that further disorganization to which it naturally tends, namely, dilatation. But, when dilatation is ascertained by its appropriate signs, if valvular unsoundness, as its cause, be excluded by the absence of endocardial murmur, and if a feeble fluttering movement of the heart be felt at every part of the præcordial region, or beyond it; and if, moreover, the constitutional habit of the man be such as to accumulate fat in all other parts, then it may be taken almost for certain that fat is especially deposited upon the heart at the expense or detriment of its muscular substance. Be it always remembered, nevertheless, that our inference, however correct it may turn out, is drawn, not directly from any express diagnostic signs, but indirectly from coincident circumstances. No murmur reaches the ear to tell us at once that the heart is fat. But we know that the heart is feebler and more capacious than natural. And we know that such, if life lasts long enough, is ultimately the condition of all fat hearts. Besides, we observe that the patient is *altogether* fat, and so we infer the probability that the heart has not escaped his constitutional peculiarity." (pp. 166-7.)

Lecture XXVI. Dr. Latham now comes to those affections of the heart which, he says, "may be usefully classed together under the name of unsoundness from disorganization." These affections consist of alterations of size and shape, and bulk and capacity—in fact, hypertrophy, atrophy, dilatation, and contraction. And Dr. Latham designates them "*as a class* by this name of 'unsoundness from disorganization,' that it may help us to keep in mind the important truth, that disease properly so called does not enter into the actual process of their formation." We confess the phraseology does not please us: we have, in the first place, no notion of *disorganization* without *disease* (unless, of course, in instances where destroying external agents act on the textures); and in the second, the application of the term *disorganization* to a state of *hypertrophy* seems to us (to use Dr. Latham's antithetic style) happily infelicitous. It is to be observed, that we do not contest the justness of the pathological notions the lecturer had in view: but object to the terms simply which he has fixed on to give signification of them. In his comments on the subject there is nothing new.

Lecture XXVIII. Here we have considerations on the fact that "unsoundness of the heart from disorganization is sometimes traceable to an accidental shock which it has sustained." Cases of three kinds are referred to rather than related;—in some, death had occurred after a certain lapse of time from the receipt of an injury in the region of the heart, or after a violent effort or a violent fit of passion, and rupture of a valve has been discovered, with superadded hypertrophy; in others death had not occurred, but palpitation, arising under the same influences as causes, had remained more or less permanent and constant, and here a similar rupture may be supposed to have occurred in the valves, or possibly some injury to the muscular structure itself; in yet other cases the clinical history was the same as in those we have just spoken of: a post-mortem examination took place, hypertrophy was discovered, but no evidence of material injury inflicted on the heart at the time of the accident, to which the symptoms of heart-disease (palpitation, painful impulse, &c.) had been confidently and uniformly referred by the patient. Now, in these latter cases a natural question arises, which Dr. Latham puts and responds to in the following manner:

"But is it quite certain in these cases that the hypertrophy and dilatation really came from a material injury done to the heart at the time of the shock? In neither of them did the heart present the visible traces of any such injury as could be conceived to proceed immediately from violence. Still I do not know that anything short of absolute disruption must necessarily leave the characteristic marks of itself ever afterwards. It is conceivable that the injury itself might not be of a permanent nature, and yet abide long enough to lay the foundation of permanent disorganization. Further it is possible that, in these same cases, causes might have been found in other parts of the body (for such it will presently appear there often really are) entitled to a share in producing what was found in the heart. Nevertheless the shock, that had been suffered in both cases, was a remarkable part of their clinical history. The patients themselves constantly ascribed to it the origin of all their malady. We cannot therefore exclude it from our consideration, and may venture, without speculating further upon what cannot be proved, to regard it as in some manner powerfully conducive to the hypertrophy and dilatation of the heart, and to the fatal event." (pp. 207-8.)

This is an interesting lecture upon a subject which has attracted comparatively little attention. It would appear to follow, from the cases referred to by the writer, that life, in case of injury to the heart's structure, has been distinctly saved by full bleeding. That quietude, moderation in sexual intercourse, &c., are indicated subsequently, when the first danger has passed, is so plain, that the fact needs not to be insisted on.

LECTURE XXIX. Conditions conducive to "organic unsoundness" of the heart are found beyond the organ itself, in parts near and in parts remote, and in the constitution at large. Thus dilatation of the aorta, as well as unnatural narrowness of the aorta, will lead to hypertrophy of the heart—both for very obvious reasons. Cases no doubt occur in which the result is not witnessed, and then counteracting influences may sometimes be detected, sometimes be matter of conjecture, and sometimes no clue to them may exist. Dr. Latham believes that dilatation of the aorta is more apt to affect the heart when it occurs as a general enlargement of the vessel over a certain space, than as an abrupt expansion in the form of a sac; and also that the nearer the alteration of form of the vessel lies to its origin, the more capable is it of producing the ill influence in question. Of the general accuracy of both these propositions we have no doubt.

Among causes exterior to the heart capable of producing its "disorganization," are certain diseases of the lungs, offering impediment to the circulation through those organs; the right cavities undergo dilatation with or without hypertrophy. But pulmonary consumption, the disease which renders useless so vast an amount, it may be, of lung, does not produce, except in rare instances, such dilatation. How comes this? Simply from the fact that various agencies are at work in the constitution at large, which bring down the amount of blood in the individual—the amount of blood requiring to pass through the lung. All this is matter of familiar knowledge; but here is an application of this familiar knowledge which, if not actually new, is well and strikingly put:

"It is remarkable in this disease [phthisis], how those symptoms which are considered to be of the most fatal omen, seem to arise out of an express provision of nature for prolonging the duration of life. The hectic perspiration, the occasional diarrhœa, the sputa, the languid powers of nutrition, all tend to keep down the current of blood to that measure which can obtain an easy passage through the lungs. On any other terms the patient would die of suffocation suddenly, and at an early period of his disease." (p. 218.)

And it follows, as well from actual observation as from reasoning, that the influence of causes seated in the lungs in producing dilatation of the right side of the heart, is best to be seen in diseases which, while they cause great impediment to the circulation, yet do not much impair the general health or the powers of nutrition.

Upon impediments arising out of morbid actions in distant parts Dr. Latham says:—

"I cannot so easily accommodate my mind to an hypothesis as to believe all that is pretended concerning them. I find depositions of lymph in the cellular texture of a limb, constituting what is called a solid œdema; I find tubercular depositions in any organ, such as the liver; I find even simple inflammations of distant parts seriously insisted upon, as if they were well-authenticated causes of disorganization of the heart, when they have happened to exist together with it. And the *theory of mechanical obstruction* is brought in confirmation of the fact. For, say the theorists, where there is inflammation, there must be spasm of the extreme vessels, and spasm is tantamount to obstruction. And again, where there is effusion or deposition of any kind, there must be pressure upon the neighbouring blood-vessels, and pressure must produce obstruction, partial or complete, according to its degree. Now, by parity of reasoning, there is no conceivable sort of morbid action in any part of the body, which may not be construed into an obstruction of the blood-vessels, and thus conjured into a possible cause of disorganization of the heart." (pp. 221-2.)

Dr. Latham turns to the influence exercised by the renal disease attended with secretion of albuminous urine, in the generation of "organic unsoundness" of the heart. Incidentally he reviews the chief morbid states of various textures and organs, which experience has taught us to connect with that renal affection;*—and he presumes it probable that the source of those various states lies in a poisoned state of the blood, an opinion which has been held by many observers, and one which is unquestionably put forward upon very plausible grounds. But Dr. Latham's remarks upon this subject are the merest generalities.

* We cannot omit this opportunity of directing the reader's attention to the researches of Dr. Taylor on the influence of Bright's disease in producing pericarditis and various other internal inflammations. These researches, contained in the paper already referred to, exhibit, to a degree for which pathologists were unquestionably not prepared by the inquiries of any preceding observer, the active power of the renal affection in generating the cardiac inflammation. But, valuable as this point of knowledge is, we consider that its establishment is but one of the least merits of Dr. Taylor's paper. We admire that paper infinitely more for its general spirit than for any of its special results,—for the devoted zeal with which truth has been sought in its pages, for the sound logic of its deductions, and its uncompromising rejection of those loose speculations which pass for wisdom with the crowd, and are known as "foolishness" by the few.

LECTURE XXX, treating of the treatment of chronic valvular unsoundness, is principally interesting from the remarks it contains upon the existence of valvular murmur in children, first *accidentally* discovered, not traceable to any attack of acute diseases, remaining for years unaltered in character, not affecting the local or general health of the individuals, or preventing them from indulging with impunity (as far as their own feelings go) in violent and athletic exercises. What is the state of things in such cases? One of the kind we have ourselves seen,—the loudest systolic endocardial murmur we ever heard, musical and sonorous, pervading the chest before and behind of a female child, aged about five years (a very model of health), had all the negative characters in respect of its history and pathological influences we have above enumerated. Dr. Latham pleads ignorance of the nature of such cases; we must be content to follow in his track.

LECTURE XXXI.—Hypertrophy of the heart—is it curable or not? Dr. Hope says he has cases which afford him "reason to believe that *nearly the whole,* who are under the age of forty, may be *radically cured,* provided the hypertrophy is exempt from complication with valvular or aortic disease, adhesion of the pericardium, softening of the heart, or other organic obstacles to the circulation; and provided, also, that the constitution is sound, and the general health tolerably good." Dr. Latham, *per contra,* says, "I must confess that in the whole course of my experience I never yet met with a single instance in which I was perfectly satisfied that hypertrophy was cured." To which of the learned doctors shall we extend our faith? Collateral evidence may help us in deciding. In the first place, the marvellous Dr. Hope gives no proof of his marvels,—he has given no single specimen of his cases; the boastful allusion recorded above is all the information we get concerning them. In the second place, it was one of the failings of Dr. Hope's mind, and one of the weaknesses of his moral nature, to believe, or to feign that he believed, himself a man of wondrous dexterity in the cure of disease,—witness the special prowess he imagined he possessed in curing acute rheumatism, and warding off harm from the membranes of the heart; as likewise his posthumous paper, wherein he appears in the amiable and scientific guise of a very heaven-sent curer of empyema! On the other hand, Dr. Latham gives no sign of undue distrust in the powers of medicine generally, and yet he unhesitatingly proclaims the incurability of the disease we speak of. Hence we are prepared to believe Dr. Latham on the point at issue; more especially as (pardon the egotism, reader,) we have yet ourselves to behold the cure of an absolutely and truly hypertrophied heart. There is a *mock* hypertrophy, so like, in some of its characters, to the real, that Dr. Latham would "find no fault with his auditors for being taken in by the counterfeit." This *mock* hypertrophy he describes graphically in these words:

"There may be violent impulse of the heart, felt not only in the præcordial region but in every part of the front of the chest upon which you lay your hand; and there may be pain in the heart, and pain and throbbing in the head; and all these may be never absent and often aggravated from time to time by accidental circumstances; and they may continue from first to last for several months or for several years, and produce in the mean while an incapacity of all useful exertion both mental and bodily: all these may be, and yet there be no hypertrophy." (pp. 248-9.)

And this morbid state is curable,—curable with variable amounts of facility in different cases, but still curable.

Dr. Latham's remarks on the necessity for caution in the employment of venesection in cases of hypertrophy (we mean the real) are good and sound. He believes that by leeching to a small extent on the præcordial region we shall be able sometimes "to overpower the most tumultuous conflict of pain and dyspnœa and nervous alarm which can be conceived," and that the plan "succeeds oftener in bringing relief than either venesection or cupping." Why this is so is not very clear, but so it is. "Beware," adds Dr. Latham, "in the management of hypertrophy of the heart, beware, above all things, of bleeding your patients into paleness and poverty of blood." And all who have witnessed the effects of that terrible combination—hypertrophy and anæmia—will echo the caution.

Lecture XXXII. Softening of the heart occurs as a part of certain general diseases,—of typhoid fever, (as shown by M. Louis, and insisted on by Dr. Stokes,) of scurvy, and of anæmia. Cure these affections—give respectively stimulants, lemon-juice, and iron—and you cure the softening also. But there is another species of softening, "secret in its beginning, and secret still in its stages and periods."

"The patients are in the decline of life, or they have forestalled the season of old age by intemperate habits. Their nervous system may be shattered: their arteries may have undergone extensive changes of structure; their livers may be enlarged, their kidneys may be granulated. All of these forms of disease may be, and some of them are sure to be, conjoined with softening of the heart, before it comes to be known and to be treated." (p. 268.)

And this softening may be accompanied with hypertrophy or atrophy, and in either case by dilatation of one or both ventricles. And all this is incurable, utterly.

Lecture XXXIII. The effects of unsoundness of the heart in its different forms upon the vascular system, venous, arterial, and capillary, form the subject of this lecture. The matter is illustrated and enforced in the usual pointed and emphatic style of the lecturer, and the whole deserves to be deeply considered by the practical physician,—but there is nothing actually new to be dwelt upon by us. The following passage, in which the author insists upon the advantages of hypertrophy of the left ventricle as a state superadded to valvular disease, seems, however, to require brief notice:

"Here it is the hypertrophy, which is the safety of the patient and enables life to go on as it does. Take away the hypertrophy and leave the injured valve, and the patient would be in a far worse state than he now is; worse with half his disease than he now is with the whole of it. The pulse would begin to flutter, the complexion would become dusky, and the lips blue, and the surface of the body mottled and patched in consequence of the blood being here and there unequally distributed or partially detained. The ventricle reduced to its common bulk would want the power needed to impel the blood steadily onwards against an extraordinary obstacle." (pp. 296-7.)

Now it appears to us that Dr. Latham's love of generalization and antithesis combined has betrayed him into the utterance of an opinion which, taken as it is uttered, stands in need of material qualification. He makes no distinction of the valve diseased, nor of the nature of the disease with which it is affected: the coexistence of systolic murmur and hypertrophy of the left ventricle is the condition spoken of. Now such a murmur may be aortic obstructive or mitral regurgitant; and it is obviously only when it is of the first kind that the occurrence of hypertrophy can conduce to the patient's well-being in the manner spoken of by Dr. Latham. For the additional power acquired by the left ventricle can only have the effect of increasing the vigour and amount of regurgitation at each systole, and so increasing all the evils which follow in the wake of insufficiency of the mitral valve, when that is the valvular affection present.

LECTURE XXXIV. Here is presented a general view of the secondary diseases which proceed from an unsound heart, and an outline of their treatment. The vast extent of their pathological range is pointedly insisted on, and the conditions limiting and enlarging the expectations of medicine in different cases duly weighed. Congestions, effusions, hemorrhages, and inflammations, are in general terms the affections which arise, and which arise in various parts and textures, under the influence of cardiac disease. Now the author derives, from his examination of the curability of these various secondary affections, a certain number of propositions, and as these propositions comprise the marrow of the whole lecture, we cannot do better than reproduce them here:

"1st. That the fact of congestions, or effusions, or hæmorrhages, or inflammations having their actuating cause in an unsound heart prohibits the possibility of their cure in *the highest sense*, and limits the expectations of medicine to their suspension, their abatement or their temporary removal.

"2dly. That the form of unsoundness in the heart furnishes a measure of calculation, how much medicine will probably be able to effect in the individual case, whether it will go to suspend or to abate or even so far as to abolish them for a time.

"3dly. That the presence or absence of coincident disease in other organs always modifies those expectations of relief by medicine, which the mere form of unsoundness in the heart itself would lead us to entertain.

"4thly. That constitutional plethora and anæmia are grave contingences when they are found in coincidence with an unsound heart, having an important bearing upon its consequences and upon our modes of treating them and upon our expectations of giving relief.

"5thly. That, besides all these intrinsic conditions, the extrinsic accident of what may be the patient's circumstances in life throws a great weight into the balance for or against the probability of relief. The man, who, having an unsound heart, must traffic with his sinews, for his daily bread, has a poor chance of benefit from medicine.

"6thly. That treatment has never more need of being favoured by opportunity than it has now." (pp. 316-17.)

LECTURE XXXV. Dr. Latham proceeds to take a more particular view of the secondary diseases springing from unsoundness of the heart. And the lungs are the organs first considered,—the considerations presented exhibit no actual novelty, but important truths, which cannot be too often or too seriously urged on the attention of the physician, are put in that striking form that cannot fail to arrest and stimulate thought.

LECTURE XXXVI brings us to the subject of atrophy caused by cardiac disease,—the treatment of which will "be simplified by keeping in mind two points,—1st, the relief which is sought by the effusion of serum; yet, 2dly, the mischief which actually follows its accumulation. Rather a subtle refinement, however, perhaps you will say, thus to claim a character of good in its design for that which inevitably terminates in evil! It is like taking a *salutary* leap into perdition."

Dr. Latham sees antitheses in things as well as employs them in words; here is a remarkable and instructive illustration of the fact:

"Among the various ways in which the fortunes in life of those, who are the subjects of an unsound heart, can modify the expectations derived from the intrinsic conditions of their disease, there is one which is very remarkable. In those who are well-off in the world, dropsy seldom arrives until the unsoundness of the heart has reached the point, at which the circulation can endure its oppression no longer without seeking relief by effusion of serum. But in those who are ill-off, dropsy often appears long before the unsoundness has reached the point at which it would naturally and necessarily take place. In the first, the injurious operations proper to the heart's unsoundness are less accelerated by accidental circumstances, and so its evil consequences and dropsy among the rest are found to tarry, until it has itself become absolutely great enough to produce them. In the second, its proper injurious operations are unavoidably quickened day by day and every hour of the day by accidental circumstances; and so a smaller disease in one case is more felt than a larger disease in the other, and its evil consequences and dropsy among the rest arrive sooner. We may well be surprised at the complete success which occasionally attends our treatment of the vast dropsical accumulations, which accompany an unsound heart. But in such cases it will be found that, but for the patient's unfortunate circumstances, there would have been no dropsy at all, the affection of the heart being not yet ripe for it." (pp. 351-2.)

Hereupon Dr. Latham tells two clinical stories, concerning "a young man" and "a little boy," who had both cardiac disease, but of whom the one recovered and the other died. But we do not see that the point sought to be illustrated by the contrast of these two cases derives any very distinct elucidation from the faint outline given of their particulars.

Lectures XXXVII and XXXVIII close the book with an account of angina pectoris. Angina pectoris is the name for an affection known only by its symptoms, and which Dr. Latham would define thus: "Pain of extreme severity passing through the chest from the sternum to the spine, arising suddenly and ceasing suddenly, and accompanied, while it lasts, with a feeling of approaching death." Dr. Latham seems to have actually seen but two fatal cases of angina pectoris; in both these organic change was found in the heart. It is well known that the theory of ossification of the coronary arteries has been frequently (in proportion to the absolute frequency of angina pectoris), proved to fail utterly: angina has killed persons with sound coronary arteries, and individuals, whose coronary arteries were as unyielding as bone, and seriously obstructed, have died of other diseases, without ever having had an attack of angina. The victims of this cardiac anguish have been found to have dilatation of the aorta, valvular disease, hypertrophy or atrophy, softening or fatty degeneration. And it has killed where "no form of disease or disorganisation whatever has been found, either in the heart or in the blood-vessels nearest to it." Hence, then, in the author's words: "we are sure of what angina is as an assemblage of symptoms; we are not sure of what it is as a disease."

And Dr. Latham's notion of the intimate nature of the affection appears in the following statement, wherein it will be perceived that he adopts the original doctrine of Heberden.*

* By the way, how came Heberden to have seen *upwards of a hundred cases* of angina? Either he must have sometimes erred in the diagnosis of the affection, or the hearts of our population have wonderfully changed since his time. Is there a living man now who has ever seen *thirty true* such cases, no matter what his age, what the extent of his practice, or what the peculiarity of his opportunities for the observation of cardiac diseases?

"'Spasm,' it has been said, 'is a mode of action in muscular structures different from, or beyond, the natural and accustomed mode.' The natural actions in all muscles, voluntary and involuntary, are unaccompanied by any conscious sensation whatever. But spasm is always accompanied with pain. And pain and spasm, wherever they are, disable the parts which they befall. Colic stops the peristaltic movements of the bowels. Cramp forbids the hands to handle, and the feet to walk. But the heart is a muscle, and its functions flow from its attributes as a muscle. Now we are in search of something in the heart which, as the concomitant of pain, may be disabling to its natural functions, and capable, according to its degree, of hindering or abolishing them altogether. This we find in spasm. In its spasm of smaller degree the heart fails to close freely upon the blood and to impel it freely into the arteries. In its spasm of greater degree it fails to project it altogether. Herein we discern an adequate explanation of the chief phenomena of angina pectoris. It is a spasm of the heart." (pp. 385-6.)

In respect of treatment of the paroxysm there are two things to be considered, the sense of dissolution and the pain. The first is to be relieved by those stimulants known to be most rapid in their effects,—and Hoffman's æther and the spiritus ammoniæ, in a drachm dose, are those recommended by Dr. Latham. Now pain is not always to be treated at all, because it passes away with the spasm, and is so short-lived that no remedy would reach it in the time. But in cases when the pain continues for a longer period than usual, a quarter of an hour or more, then opium (a drachm of laudanum) must be given with the æther.

And now must we bring to a conclusion our notice of these Lectures; the series now reviewed amply sustains the reputation of the first; and to set down our general opinion of their merit, would be but to repeat the strong eulogia with which we conveyed our impression of the signal excellence of the former volume.

Before we finally lay down our pen, we must write a few more words on the literary character of this work. In our notice of the first volume, we gave to Dr. Latham's style the highest general commendation, while we made some exceptions to its antithetical quaintness and mannerism. The present volume is exactly like its predecessor in every respect,—indeed, too like; as it is impossible not to feel that, with all its excellencies, the style is rather artificial and monotonous, and therefore somewhat tiresome. At the same time, we must add, that it is only when regarded in relation to the general high cast of composition in these volumes, that Dr. Latham's style can be pronounced even partially defective. Compared with that of the great majority of medical books, the language appears almost perfect, and compared with the very best, it may fairly contend for the first place. The language is, indeed, throughout—not merely in every chapter or page, but in every sentence—faultlessly accurate, and at the same time, polished in the highest degree. In this respect, and, indeed, in other respects also, the volumes before us are well entitled to the name of classical; and they will, we doubt not, take a place among the very small number of medical books that will be prized and read by men of other generations.

